# Juvenile open-angle Glaucoma associated with Leber’s hereditary optic neuropathy: a case report and literature review

**DOI:** 10.1186/s12886-018-0980-2

**Published:** 2018-12-17

**Authors:** Yun-Hsuan Lin, Nan-Kai Wang, Ling Yeung, Chi-Chun Lai, Lan-Hsin Chuang

**Affiliations:** 1 Department of Ophthalmology, Chang-Gung Memorial Hospital, 222 Mai-Chin Rd, Keelung, 204 Taiwan (Republic of China); 2 Department of Ophthalmology, Chang-Gung Memorial Hospital, Linkou, Taiwan; 3 College of Medicine, Chang Gung University, Taoyuan, Taiwan

**Keywords:** Leber’s hereditary optic neuropathy, Juvenile open-angle glaucoma, Mitochondria

## Abstract

**Background:**

Leber’s hereditary optic neuropathy (LHON) is a maternally inherited recessive disease rarely complicated with glaucoma. We conducted a clinical and genetic retrospective case series to describe three cases of juvenile open-angle glaucoma (JOAG) and an ND4 m11778G > A mitochondrial DNA (mtDNA) mutation, which is pathognomonic for LHON.

**Case presentation:**

Patient 1 was a 16-year-old boy diagnosed with bilateral JOAG and high myopia. His intraocular pressure (IOP) was poorly controlled with the use of full topical anti-glaucoma medications. His best-corrected visual acuity (BCVA) decreased gradually over 5 years. Fundoscopic examination revealed bilateral enlarged disc cupping of the optic nerves with sectorial excavation and reduction of the neural rim in the left eye. His visual field (VF) was characterized by bilateral progressive central scotoma. Pattern visual evoked potentials (VEPs) and pattern electroretinograms (ERGs) showed extinguished responses in both eyes. Because of the non-specific visual field findings and the optic neuropathy disclosed by the pattern VEPs and pattern ERGs, we arranged a genetic test for the patient, which revealed an m11778G > A mtDNA mutation. Patient 2, the younger brother of Patient 1, was a 15-year-old boy who had been diagnosed with bilateral JOAG in 2010. The BCVA of both eyes remained at 1.0 during the follow-up period. Fundoscopic examination revealed bilateral mildly paled optic disc with enlarged cupping and reduction of the neural rim. The pattern ERG revealed a decreased N95 amplitude bilaterally. The genetic test revealed an m11778G > A mtDNA mutation. Patient 3 was a 35-year-old man with bilateral JOAG. His BCVA decreased gradually over 10 years. Fundoscopic examination revealed paled optic disc with enlarged disc cupping and reduction of the neural rim in both eyes. The pattern ERG revealed a decreased N95 amplitude bilaterally. The genetic test revealed an m11778G > A mtDNA mutation.

**Conclusions:**

This case series describes three patients with concomitant occurrence of JOAG and LHON. These two diseases may have a cumulative effect on oxidative stress and retinal ganglion cell death with the rapid deterioration of vision, which may occur during adolescence.

## Background

Leber’s hereditary optic neuropathy (LHON) is a maternally inherited recessive disease caused by a mutation in the mtDNA. In 1858, Dr. Albrecht von Graefe proposed the first mitochondrial disease attributed to a point mutation. Dr. Theodore Leber reported 15 patients with this disease years later; the disease was therefore named after him [1, 2]. The selective degeneration of retinal ganglion cells (RGCs), which are sensitive to mitochondrial dysfunction, leads to vision loss [3]. LHON develops mostly in young adult males with a mean age of onset between 18 years and 35 years. The affected individuals typically present unilateral acute/subacute, painless, and central/cecocentral visual loss. Six to 8 weeks later, the fellow eye becomes involved [4, 5]. Abnormal cupping of the optic nerve head can be found by clinicians. The appearance in the atrophic stage of LHON is similar to that in glaucoma patients. In addition, an association between normal-tension glaucoma and LHON has also been described [4, 6]. Although retinal ganglion cell death is evident in both LHON and glaucoma, the disease course and pathophysiology of these two diseases are different. A slow and progressive disease course and the presence of elevated intraocular pressure (IOP) are commonly seen in glaucoma patients, while an acute onset disease course and point mutations in mtDNA are seen in LHON. Few studies have demonstrated the simultaneous occurrence of these two diseases. In this case series, we describe the concomitant occurrence of juvenile open-angle glaucoma (JOAG) and LHON in 3 patients, 2 of whom are siblings.

This study has a retrospective design. The medical records of 3 patients who presented with JOAG and LHON were reviewed. Patient records were reviewed for demographic information, best-corrected visual acuity (BCVA) with the Snellen chart, IOP at disease onset, nadir and last follow-up clinic visit, visual field (VF), and gonioscopic, pachymetric, and fundoscopic examinations. Due to the atypical visual field defect pattern in these patients, electrophysiological testing and genetic testing were arranged and recorded as well. This study was approved by the Institutional Review Board of the Chang Gung Medical Foundation.

## Case presentation

### Case 1

**Fig. 1:**
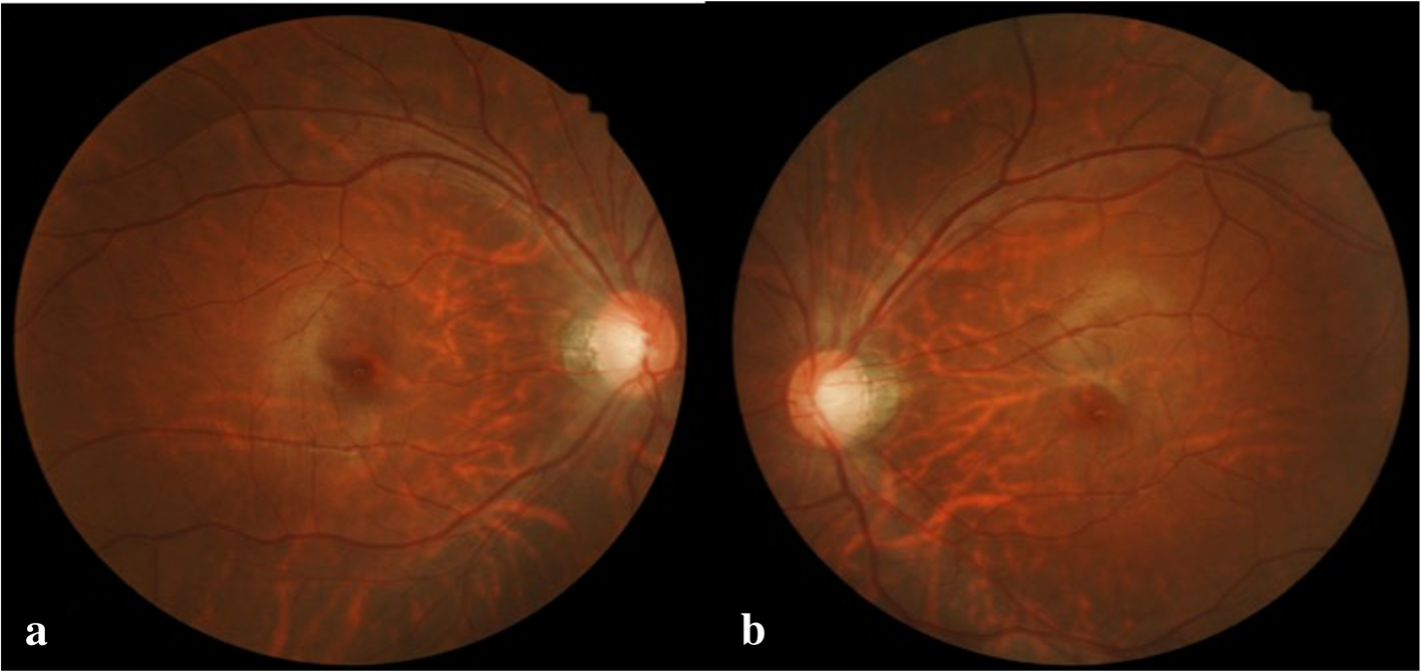
Fundoscopic photographs of the right eye (**a**) and left eye (**b**) of Patient 1. The fundoscopic photographs of Patient 1 revealed bilateral enlarged disc cupping of the optic nerves with sectorial excavation and reduction of the neural rim in the left eye

**Fig. 2:**
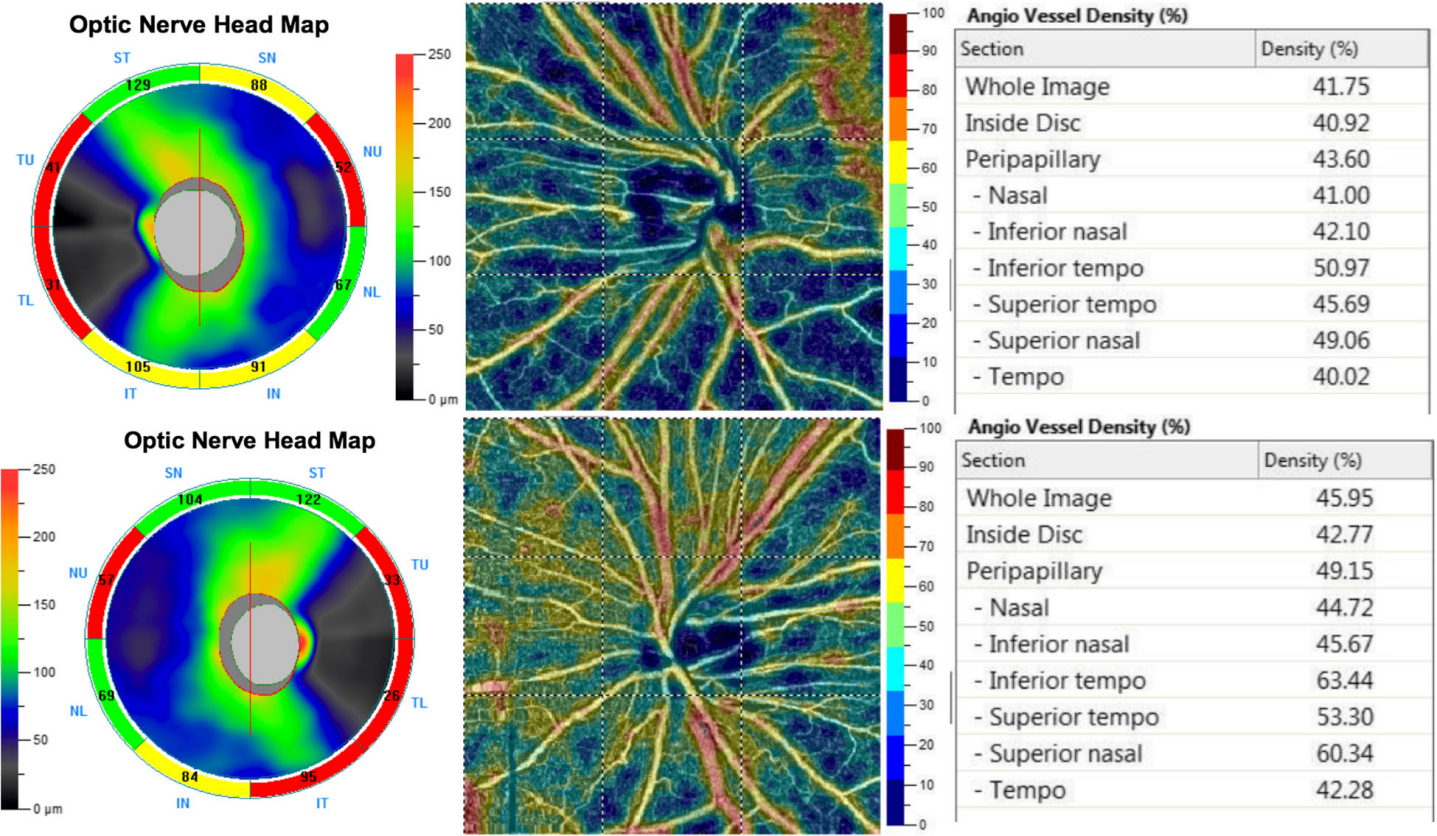
Optical coherence tomography angiography and vessel density results of the right eye (upper) and left eye (lower) of Patient 1. The optical coherence tomography angiography of Patient 1 revealed retinal nerve fiber layer thinning over the upper and lower temporal quadrants and upper nasal quadrant of the right eye and retinal nerve fiber layer thinning at the temporal quadrant and upper nasal quadrant of the left eye. The peripapillary vascularity of the capillary was 43.6% for the right eye and 49.15% for the left eye. Both eyes revealed a decrease in the sectoral division of the temporal regions

**Fig. 3:**
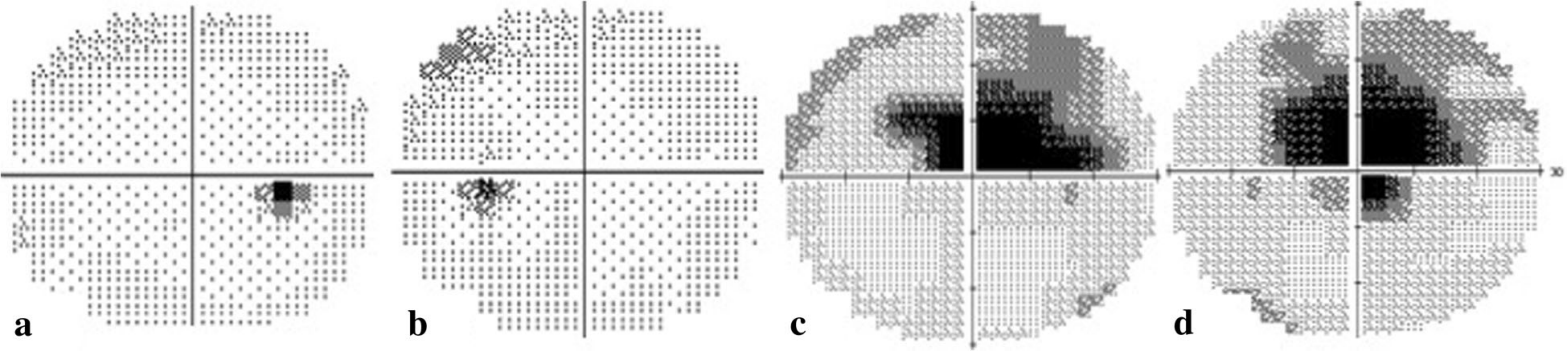
The visual fields of the right eye (**a**) and left eye (**b**) of Patient 1 in 2013 and the visual fields of the right eye (**c**) and left eye (**d**) of Patient 1 in 2016 The visual field (30–2 SITA standard) was characterized by bilateral progressive central scotoma in the eyes from 2013 to 2016. *SITA: Swedish interactive thresholding algorithm.

**Fig. 4:**
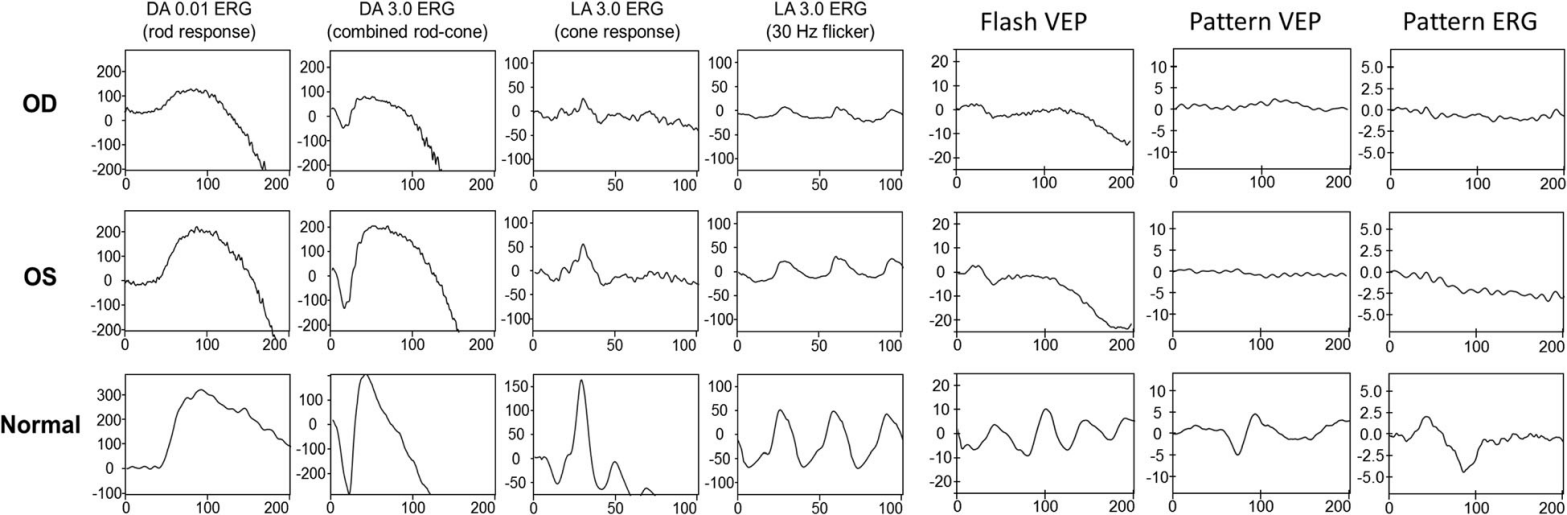
The electroretinogram (ERG), pattern visual evoked potential (VEP) and pattern ERG results of Patient 1. The full-field ERG was subnormal for both eyes, and there were more decreased amplitudes in the right eye than there were in the left eye, but there was no obvious implicit time delay noted. The pattern VEP and pattern ERG results showed bilateral extinguished responses in the eyes. *ERG: electroretinogram; VEP: visual evoked potential

A 16-year-old boy without systemic disease had come to our attention in October 2012 with a diagnosis of bilateral JOAG and high myopia (− 8.0D). Although he was treated with dorzolamide 2%, latanoprost 0.005%, brimonidine 0.2%/timolol 0.5% fixed combination, and oral acetazolamide 500 mg/day at another hospital, his IOP was approximately 25 mmHg and was poorly controlled in both eyes by full medication. Several months before this visit, he had ceased treatment with anti-glaucoma medication. His BCVA was 20/20 in both eyes in 2012, though it gradually deteriorated to 20/400 in both eyes in 2017. Gonioscopy revealed a normal iridocorneal angle; pachymetric measurements were 611 μm in the right eye and 614 μm in the left eye. The axial length of the right eye was 27.56 mm and that of the left eye was 27.46 mm. Fundoscopic examination revealed bilateral enlarged disc cupping of the optic nerves with sectorial excavation and reduction of the neural rim in the left eye (Fig. 1). Optical coherence tomography angiography (OCTA) revealed retinal nerve fiber layer (RNFL) thinning over the temporal upper and lower quadrants and nasal upper quadrant of the right eye and RNFL thinning at the temporal quadrant and nasal upper quadrant of the left eye (Fig. 2). The vascularity of the peripapillary capillaries was 43.6% in the right eye and 49.15% in the left eye. Both eyes revealed a decrease in the sectoral division of the temporal regions (Fig. 2). A VF of 30–2 Swedish interactive thresholding algorithm standard (30–2 SITA standard) was characterized by progressive central scotoma in both eyes (Fig. 3). Due to the presence of bilateral progressive central scotoma, further examinations were arranged. The electroretinogram (ERG) result was subnormal in both eyes, and there were more decreased amplitudes in the right eye than there were in the left eye. However, no obvious implicit time delay was noted. The pattern visual evoked potential (VEP) and pattern ERG showed extinguished responses in both eyes (Fig. 4). A brain MRI revealed an incidental finding of a 0.8 mm non-enhancing nodule in the pituitary gland. Furthermore, the genetic test revealed an ND4 m11778G > A mtDNA mutation, which is pathognomonic for LHON. High-dose ubidecarenone (240 mg/day) was prescribed by the neurologist. However, under high-dose ubidecarenone therapy, the patient’s IOP was still poorly controlled and his visual acuity gradually decreased to less than 20/400 in both eyes after one year of ubidecarenone treatment.

### Case 2

**Fig. 5:**
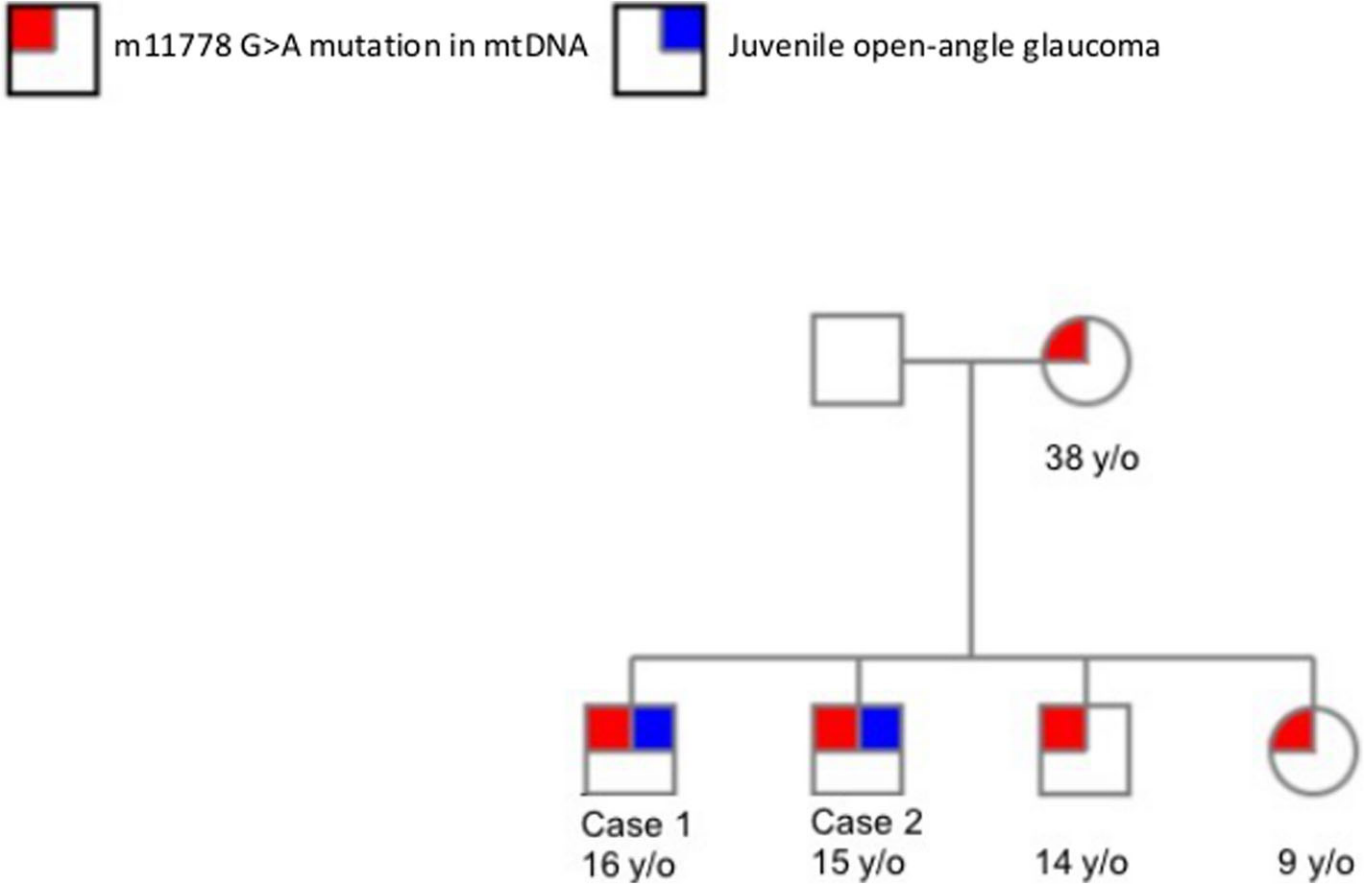
The family pedigrees of Patient 1 and Patient 2. Patient 1 and Patient 2 were both diagnosed with JOAG and an mtDNA m11778 G > A mutation. The genetic tests revealed an mtDNA ND4 m11778G > A mutation in the younger brother, younger sister, and mother. *mtDNA: mitochondrial DNA; y/o: year-old

**Fig. 6:**
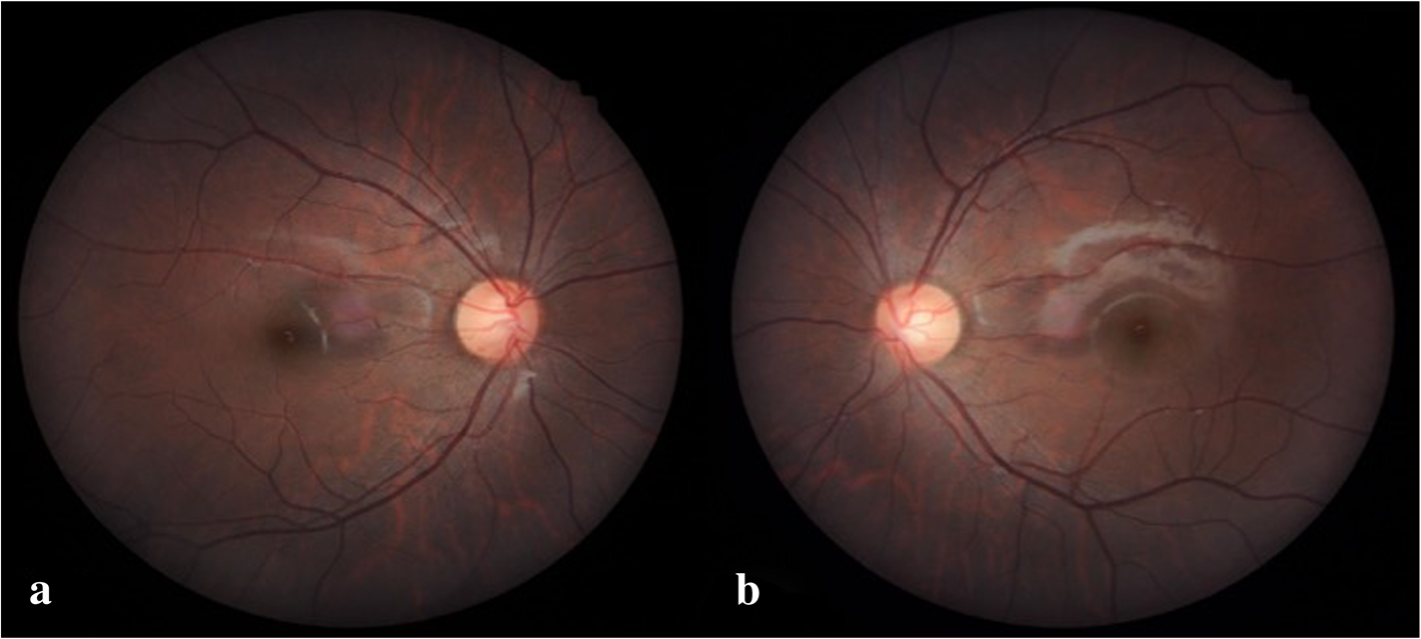
Fundoscopic photographs of the right eye (**a**) and left eye (**b**) of Patient 2. Fundoscopic photographs of Patient 2 revealed bilateral mildly paled optic disc with enlarged cupping and bilateral reduction of the neural rim in the eyes

**Fig. 7:**
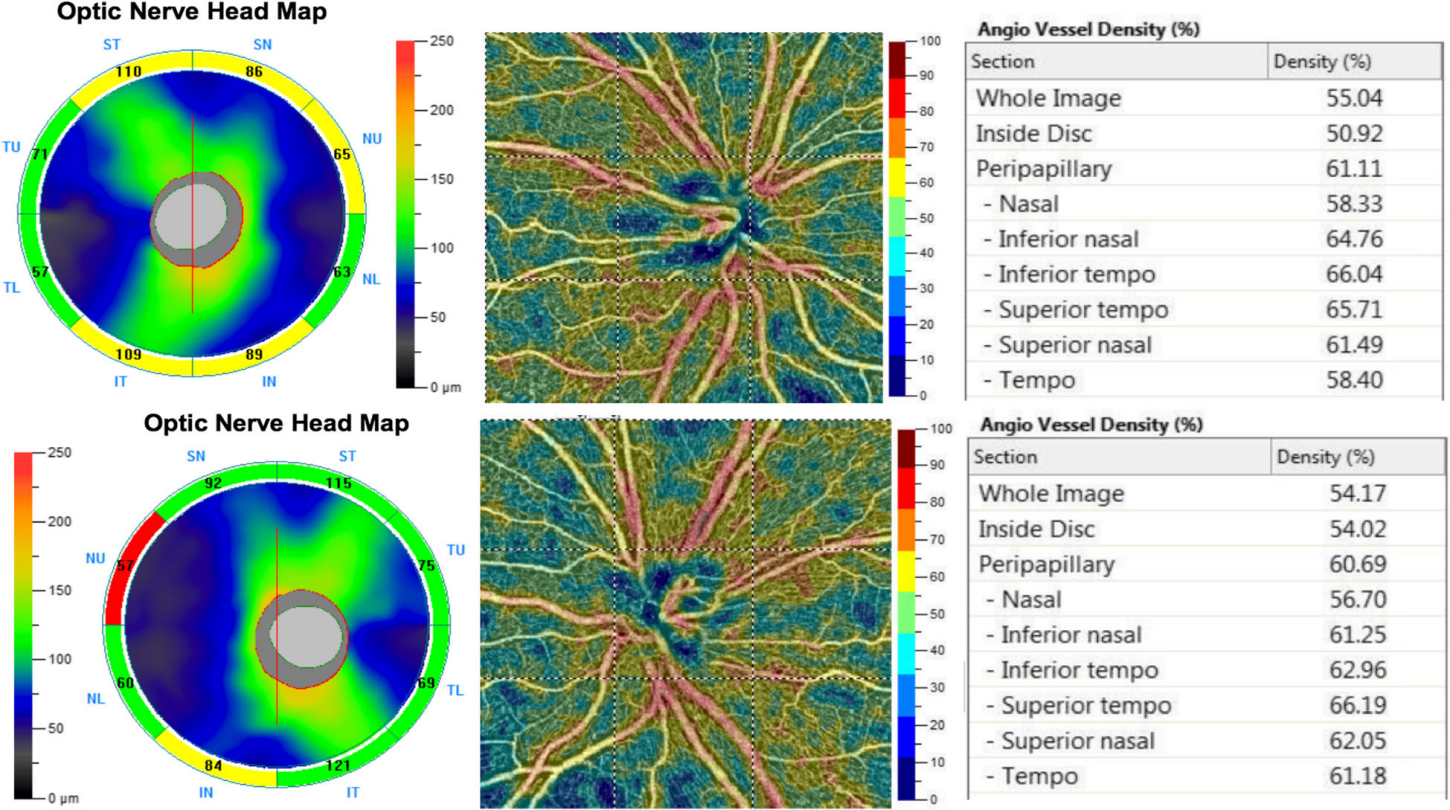
Optical coherence tomography angiography and vessel density results of the right eye (upper) and left eye (lower) of Patient 2. Optical coherence tomography angiography of Patient 2 revealed retinal nerve fiber layer thinning at the upper nasal quadrant of the left eye

**Fig. 8:**
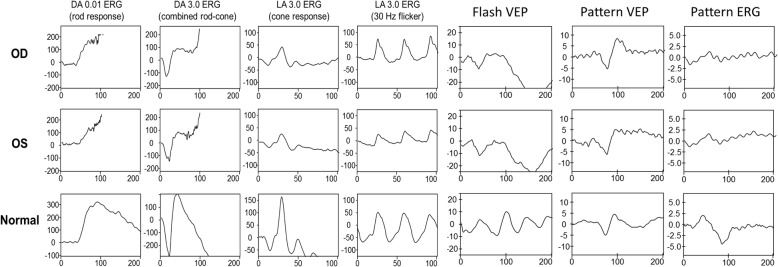
The electroretinogram (ERG), pattern visual evoked potential (VEP) and pattern ERG results of Patient 2. The pattern VEP showed no delay, and the pattern ERG revealed decreased amplitudes for N95 in both eyes. *ERG: electroretinogram; VEP: visual evoked potential

A 15-year-old boy, the first younger brother of Patient 1 who was diagnosed with bilateral JOAG in 2010, attended our clinic in October 2012. He denied having systemic disease, but he had a family history of glaucoma and LHON (Fig. 5). After taking medication, including dorzolamide 2%/timolol 0.5% fixed combination, latanoprost 0.005%, and brimonidine 0.15%, IOP was controlled in both eyes. His BCVA of both eyes remained at 20/20 during the follow-up period. Gonioscopy revealed a normal iridocorneal angle, and the pachymetric measurements were 592 μm in both eyes. Fundoscopic examination revealed bilateral mildly paled optic disc with enlarged cupping and reduction of the neural rim in both eyes (Fig. 6). OCTA revealed RNFL thinning at the nasal upper quadrant of the left eye (Fig. 7). The VF (30–2 SITA standard) was normal in both eyes during the follow-up period. The pattern VEP showed no delay, and the pattern ERG revealed decreased N95 amplitudes in both eyes (Fig. 8). The genetic test revealed an ND4 m11778G > A mtDNA mutation, which is pathognomonic for LHON. High-dose ubidecarenone(240 mg/day) was prescribed as well. During the course of high-dose ubidecarenone treatment, the patient’s IOP, visual acuity, and visual field remain stable.

### Case 3

**Fig. 9:**
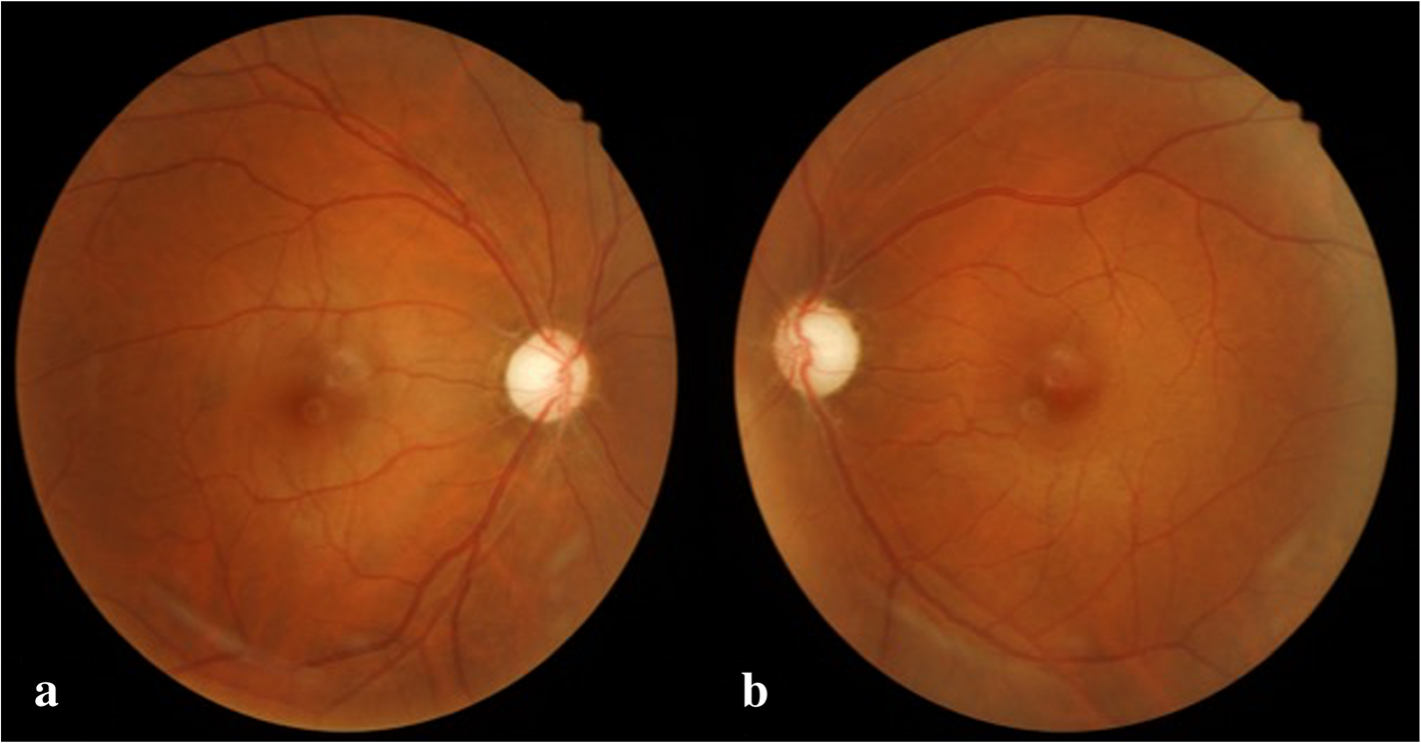
Fundoscopic photographs of the right eye (**a**) and left eye (**b**) of Patient 3. Fundoscopic photographs of Patient 3 revealed paled optic disc with enlarged disc cupping of the optic nerves and sectorial excavation and reduction of the neural rim in both eyes

**Fig. 10:**
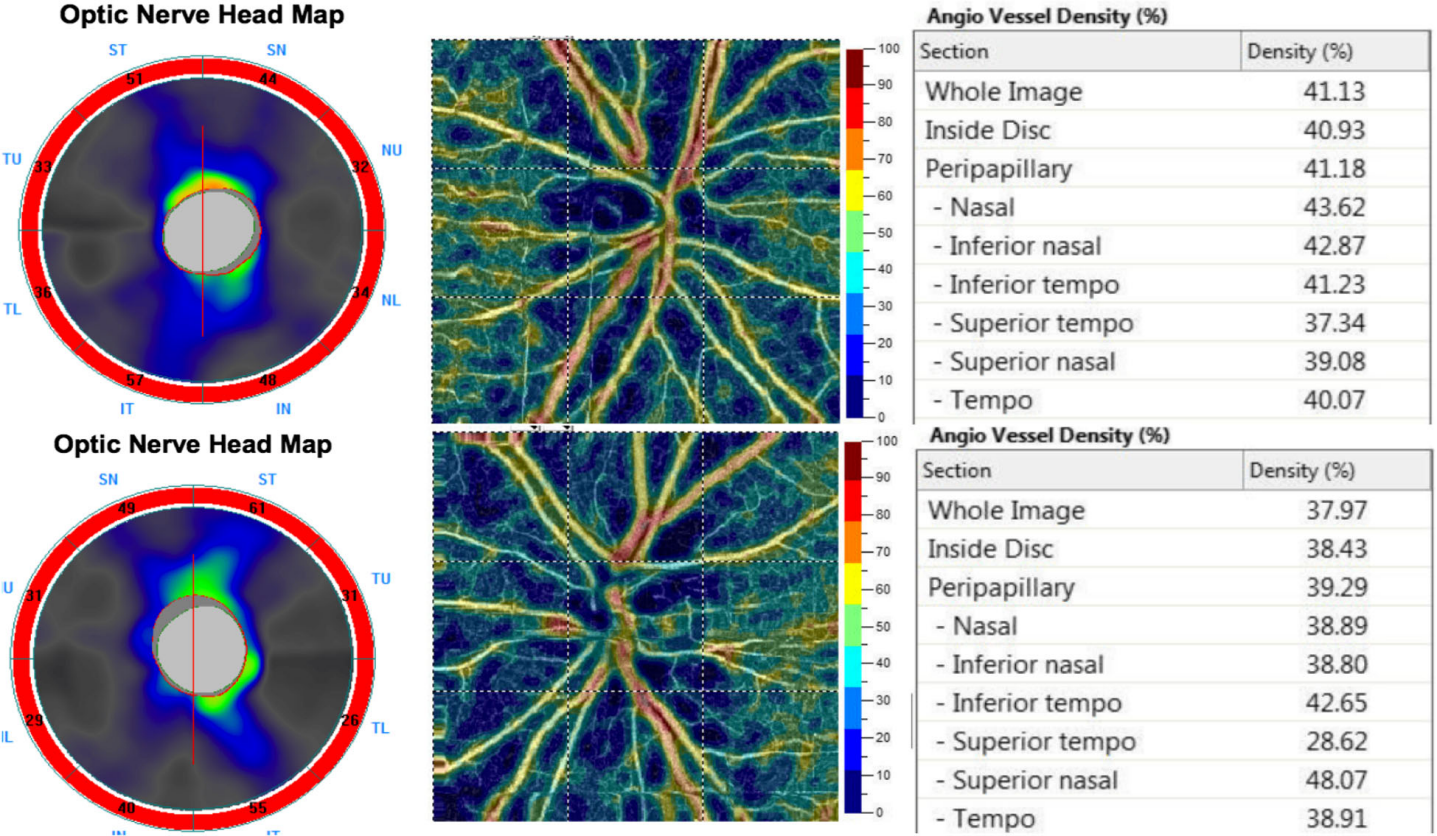
Optical coherence tomography angiography and vessel density results of the right eye (upper) and left eye (lower) of Patient 3. Optical coherence tomography angiography of Patient 3 disclosed diffuse RNFL thinning and a bilateral decrease in the peripapillary vascularity in the eyes

**Fig. 11:**
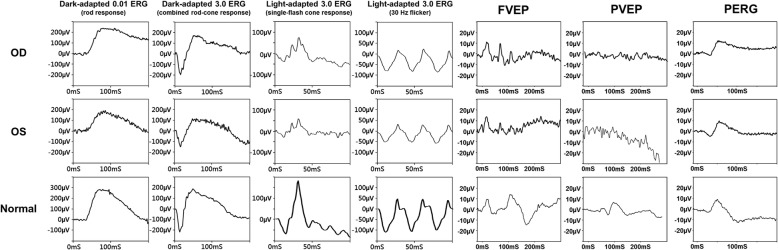
The electroretinogram (ERG), pattern visual evoked potential (VEP) and pattern ERG results of Patient 3. The ERG result was subnormal in both eyes, and the pattern ERG revealed decreased amplitudes for N95 in both eyes. *ERG: electroretinogram; VEP: visual evoked potential

A 35-year-old man without systemic disease first attended our clinic in August 2004 for bilateral JOAG. He denied having a family history of glaucoma, but his uncle had been diagnosed with LHON. When he was undergoing therapy with timolol 0.5%, his IOP was approximately 20 mmHg in both eyes. His BCVA gradually decreased from 20/200 in both eyes in 2006 to counting fingers at 25-30 cm in both eyes in 2016. Gonioscopy revealed a normal iridocorneal angle; pachymetric measurements were 561 μm in the right eye and 563 μm in the left eye. Fundoscopic examination revealed paled optic disc with enlarged disc cupping of the optic nerves with sectorial excavation and reduction of the neural rim in both eyes (Fig. 9). OCTA disclosed diffuse RNFL thinning and a decreased peripapillary vascularity in both eyes (Fig. 10). The VF (30–2 SITA standard) was characterized by progressive central scotoma in both eyes. The ERG was subnormal in both eyes, and the pattern ERG revealed decreased N95 amplitudes in both eyes (Fig. 11). The genetic test revealed an ND4 m11778G > A mtDNA mutation, which is pathognomonic for LHON.

## Discussion and conclusions

This case series describes three patients with an ND4 m11778G > A mtDNA mutation who had the concomitant occurrence of JOAG and LHON. Among them, Patient 1 and Patient 2 are brothers. In 2013, Nucci et al. had described a woman with open-angle glaucoma progression that was associated with Leber’s hereditary optic neuropathy (LHON). This patient also had the ND4 m11778G > A mtDNA mutation [7]. Approximately 90% of patients with LHON carry one of three mtDNA point mutations, which are located at the nucleotide positions 3460, 11,778, and 14,484 [8]. Among them, the most common point mutation, which accounts for 70% of all cases, is that at position 11,778, which is that identified in these cases [8]. The 14,484 and 3460 mutations account for approximately 14 and 13%, respectively, of the total number of cases [9, 10]. Previous studies have demonstrated that LHON predominantly affects males (80–90% of cases) [8]. In addition, 60% of LHON patients are aware that they have a family history of LHON, although 40% of them deny having a known family history [11].

The onset of symptoms in LHON typically occurs at young ages, but there have been few reports of symptom onset from 2 years to 87 years of age [8, 12]. The clinical presentation of LHON is acute or sub-acute, unilateral, and painless visual loss followed by the involvement of the other eye within 1–2 months. Visual acuity is typically worse than 20/200 or light perception [13, 14]. Visual field defects are typically central or cecocentral scotoma, and dyschromatopsia is often present early in the disease progression [15]. Acute funduscopic abnormalities seen in LHON include swelling of the nerve fiber layer around the disc, hyperemia of the optic nerve head, circumpapillary telangiectasia, tortuous engorged retinal vessels, and the absence of disc leakage on fluorescein angiography. Following the acute stage of the disease, the tortuosity of the retinal vessels improves, the telangiectatic vessels resolve, and optic nerve pallor (without swelling) develops [16, 17]. The funduscopic findings of the patients in the current case series revealed that they were all in the chronic stage and had a pallor optic disc and enlarged disc cupping.

Peripapillary retinal nerve fiber layer (RNFL) and vessel density changes seen in LHON can be characterized and quantified by OCTA. Impaired mitochondrial function in LHON leads to early axonal swelling of the RGCs. Previous studies has observed that, in OCTA, the RNFL thickens first in the temporal and inferior quadrants, and then in the superior and nasal quadrants in the acute phase [18]. A few months later, the thickness of RNFL decreased during the chronic phase [19], which was fully demonstrated in the OCTA findings of Patient 1 and Patient 3 in the current study. Additionally, changes in the peripapillary vascularity of the capillaries were also observed in our cases. The sectoral division changes of the RNFL and peripapillary vascularity of the capillaries in our cases were not typical changes seen in primary open-angle glaucoma patients.

Visual evoked potentials (VEPs) reflect optic nerve fiber degeneration; pattern ERGs detect RGC loss and are often abnormal in patients with LHON. Findings for concomitance are different from that for typical glaucoma or LHON. In Patient 1, the VEP and pattern ERG both showed extinguished responses in both eyes. The pattern ERGs of Patient 2 and Patient 3 revealed decreased N95 amplitudes in both eyes. Salomao et al. reported that in a Brazilian family the pattern-reversal VEP showed prolonged latencies, decreased amplitudes, or extinguished responses in three of the affected members [20]. Dorfman et al. presented two brothers with LHON, and the earliest detected VEP abnormalities were abnormal pattern and increase latency. In addition, the VEP latency further prolonged with disease progression [21]. Ziccardi et al. presented patients who also had an prolonged VEP latency, decreased VEP amplitude, and a decreased ERG amplitude in N50–N95 [22].

Magnetic resonance imaging (MRI) finding in LHON patients is usually normal [23]. However, there are reports of patients who had an increased signal in the optic nerve on T2-weighted fast spin echo and short-time inversion recovery sequences in the chronic atrophic stage [24, 25]. Optic nerve enhancement on post-contrast MRI images has been demonstrated in some case reports. That suggests that the LHON may have an inflammatory component in its pathogenesis [26].

Affected LHON patients typically have poor visual prognosis. Permanent vision loss was observed in most affected individuals, and only few studies have reported spontaneous visual recovery [27]. The partial visual recovery rate of patients with the 14,484 mutation was 37–58%. Individuals with the 3460 mutation have an approximately 20% partial recovery rate. The 11,778 mutation is associated with the lowest recovery rate, with a partial recovery rate of 4% [5, 11, 28, 29].

Recent studies in LHON cybrids have shown that the mtDNA mutations reduce the rate of respiration and oxygen consumption with increased superoxide production [30]. Since ATP production is an essential task of mitochondria, they play vital roles for the cell, including in the detoxification of reactive oxygen species (ROS), iron metabolism, amino acid biosynthesis, fatty acid oxidation, and cellular apoptosis. As highly ATP requiring tissue, the RGCs are sensitive to metabolic insult from mitochondrial dysfunction in LHON [3]. On the other hand, Sundaresan observed that 50% of patients with POAG have pathogenic mtDNA mutations, which was demonstrated by massive parallel sequencing [31]. Mitochondrial dysfunction can lead to the accumulation of ROS, which may affect the cellularity of the trabecular meshwork and impair aqueous drainage, leading to an elevated IOP [32, 33]. In previous studies, the lamina cribrosa cells from glaucoma donors were shown to have higher levels of ROS and compromised anti-oxidant potential compared with normal donors, suggesting that mitochondrial dysfunction and accumulation of oxidative stress are important in the pathogenesis of POAG [32, 34]. Additionally, Nucci et al. reported an atypical LHON (late adult female) and hypothesize that glaucoma had a superimposed cumulative effect on the oxidative stress in LHON patients, causing the RGCs damage and leading to the clinical manifestations of LHON [7].

In conclusion, this case series describes the concomitant occurrence of JOAG and LHON. Glaucoma may have cumulative effect on oxidative stress and RGCs death in LHON patients, leading to the rapid progression of visual deterioration, which may occur during adolescence with untypical findings in functional examinations.

## Abbreviations

BCVA: Best-corrected visual acuity
ERGs: Electroretinograms
IOP: Intraocular pressure
JOAG: Juvenile open-angle glaucoma
LHON: Leber’s hereditary optic neuropathy
MRI: Magnetic resonance imaging
mtDNA: Mitochondrial DNA
OCTA: Optical coherence tomography angiography
RGCs: Retinal ganglion cells
RNFL: Retinal nerve fiber layer
ROS: Reactive oxygen species
VEPs: Visual evoked potentials
VF: Visual field
